# Association between preoperative serum insulin levels and lymph node metastasis in endometrial cancer—a prospective cohort study

**DOI:** 10.1002/cam4.1391

**Published:** 2018-03-13

**Authors:** Nan Mu, Mei Dong, Chunyan Liu, Xiuli Wang, Jianglin Cong, Liqian Wang, Xiaojie Wang, Ishan Lakhani, Xia Liu, Jianqing Hou, Shaoguang Wang, Gary Tse

**Affiliations:** ^1^ Department of Gynecology and Obstetrics Yantai Yuhuangding Hospital Affiliated to Qingdao University Yantai Shandong Province China; ^2^ Department of Cardiology Yantai Yuhuangding Hospital Affiliated to Qingdao University Yantai Shandong Province China; ^3^ Department of Medicine and Therapeutics Faculty of Medicine Chinese University of Hong Kong Hong Kong, SAR China; ^4^ Li Ka Shing Institute of Health Sciences Faculty of Medicine Chinese University of Hong Kong Hong Kong, SAR China

**Keywords:** Endometrial cancer, insulin, lymph node metastasis

## Abstract

Endometrial cancer is a common gynecological malignancy in developed countries. Insulin has been identified as a risk factor for endometrial cancer. However, whether insulin levels are related to the risk of lymph node metastasis (LNM) in endometrial cancer is unknown. We conducted a prospective cohort study in a regional hospital to examine the relationships between insulin levels and risk of LNM in premenopausal and postmenopausal women. A total of 668 patients were recruited. Of these, 206 were premenopausal (mean age: 42.01 ± 10.17) and 462 were postmenopausal (mean age: 62.13 ± 13.85). The incidence of LNM in both premenopausal and postmenopausal groups was comparable at 7% and 8%, respectively. In premenopausal women, multivariate logistic regression demonstrated that insulin levels (OR: 2.11, 95% CI: 1.48–2.85, *P* < 0.05) were significant predictors of LNM risk. In the same group, insulin levels remained significant predictors of LNM risk (cut‐off: 10.48 *μ* IU/mL) when adjusted for body mass index (BMI) (OR: 3.51, 95% CI: 1.42–5.98; *P* < 0.05) or for waist‐to‐hip ratio (WHR) (OR: 1.87, 95% CI: 1.08–2.66; *P* < 0.05). Similarly, in postmenopausal women, multivariate logistic regression showed that insulin levels (OR: 1.99, 95% CI: 1.30–2.89; *P* < 0.05) also significantly predicted LNM risk. This relationship was maintained even after adjustment for BMI (cut‐off: 7.40 *μ* IU/mL, OR: 1.99, 95% CI: 1.01–3.12, *P* < 0.05) or for WHR (cut‐off: 10.15 *μ* IU/mL, OR: 1.61, 95% CI: 1.04–2.35; *P* < 0.05). Insulin levels are significantly associated with LNM risk in both premenopausal and postmenopausal women with endometrial cancer. Further prospective studies are needed to examine a potential causal relationship and determine whether its use can offer incremental value for risk stratification in this patient population.

## Introduction

Endometrial cancer is a common gynecological malignancy in developed countries. In 2017, an estimated 61,380 newly diagnosed cases and 10,920 deaths occurred due to this condition in the United States alone 1. About 85% of cases are endometrioid adenocarcinoma, which is characterized by a slow development process and a relatively good prognosis 2. Since symptoms such as abnormal vaginal bleeding usually present earlier, most patients receive their diagnoses at an early stage 3. Moreover, in approximately 75% of patients, the malignancy is confined to the uterus at the time of the diagnosis 4. Pelvic and para‐aortic lymph node metastasis has been observed in 9% and 5% of affected patients, respectively 5, 6. According to International Federation of Gynecology and Obstetrics (FIGO) staging system, this lymphatic involvement often indicates a poor prognosis 7.

To date, surgery is the primary treatment for endometrial cancer. According to the FIGO 2009 document 7, the operation procedure should include a hysterectomy, bilateral salpingo‐oophorectomy, pelvic and para‐aortic lymphadenectomy, and peritoneal cytology for staging. Except for the removal of metastatic lesions, systematic lymph node resection can decrease pelvic recurrence and provide guidance for therapeutic strategy 8, 9. However, two randomized control trials suggest otherwise, having demonstrated a limited benefit for those who have undergone a systematic lymphadenectomy 10, 11. The most frequently observed complications in such a procedure include ileum obstruction, extended ileus, deep venous thrombosis, and lymphocyst formation 12, 13. At this juncture, there is little consensus regarding the routine use of lymphadenectomy in the treatment of endometrial cancer. Furthermore, although there is no singular definitive method for evaluating the extent of lymph node metastasis preoperatively, methods including magnetic resonance imaging, computed tomography, and B‐mode ultrasonography all possess diagnostic value 14. However, these diagnostic methods are only moderately successful, thereby necessitating the need for additional biomarker testing, such as the examination of serum cancer antigen‐125 (Ca‐125) levels, which may, in turn, provide incremental value for risk stratification. Unfortunately, this is neither a sufficiently sensitive test, as only a small proportion of endometrial cancer patients have increased Ca‐125 levels 15, nor a specific test, as Ca‐125 levels are also elevated in other conditions such as inflammatory diseases and atrial fibrillation 16.

More recent studies have focused on the role of metabolic parameters in aiding risk stratification in endometrial cancer 17, as it is an estrogen‐dependent malignancy. Insulin resistance has been identified as a significant risk factor 18 for endometrial cancer. Insulin resistance refers to a condition in which target organs show a reduced responsiveness to insulin. This results in a hyperactivity of pancreatic cells, which attempt to compensate for the attenuated sensitivity, leading to hyperinsulinemia 19. Epidemiological evidence has shown a correlation between insulin resistance and endometrial cancer. The mechanisms supporting this association can be explained primarily through both the direct and indirect roles of insulin in endometrial cancer development. Directly, insulin binding to its cognate receptor induces the activation of the phosphatidylinositol‐4,5‐bisphosphate 3‐kinase (PI3K)/Akt and Ras/mitogen‐activated protein kinase (MAPK) pathways, which trigger the activation of proteins involved in cell cycle regulation. With cancer, signaling in these pathways is often dysfunctional and hyperactive 20, and can in turn precipitate in cell proliferative and anti‐apoptotic effects that collectively promote endometrial cancer development 18. Insulin indirectly induces the pathogenesis of endometrial cancer by inhibiting the synthesis of sex hormone‐binding globulin (SHBG), which normally binds to androgens and estrogens. With hyperinsulinemia, this inhibition, in turn, increases the levels of free androgens (which can then be converted to estrogens in peripheral tissues) and estrogens. As suggested by the “unopposed estrogen” hypothesis, without sufficient protection from progesterone, the resulting elevated plasma estrogen levels induce cell proliferation in the endometrium, thereby promoting carcinogenesis 21. The other possible mechanistic links between insulin and endometrial cancer will be further elaborated upon later.

Our group has reported that insulin was associated with a dose‐dependent increase in endometrial cancer risk 21. However, there have been little data on the association between insulin levels and the risk of lymph node metastasis. Therefore, we conducted a case–control study in China to evaluate whether insulin is a preoperative indicator of lymph node metastasis in endometrial cancer.

## Subjects and Methods

### Patient selection

This study was approved by the Local Ethics Committee of Yantai Yuhuangding Hospital in Shandong, China (Approval Number: E2009033). Consecutive patients with a histological diagnosis of endometrial cancer between January 2010 and December 2016 at the Yantai Yuhuangding Hospital in Shandong, China, were enrolled. All subjects provided written informed consent. Exclusion criteria included the use of steroid hormones within the past 12 months of the study.

A total of 668 patients, including 206 premenopausal and 462 postmenopausal women, were included in our study. All patients received standard staging operations including a systematic lymphadenectomy. All operations were performed by the same surgeons. All specimens were diagnosed histologically by two independent, experienced pathologists. Patients were divided according to menopause status into a premenopausal group and a postmenopausal group. Within each group, patients were further divided into a lymph node metastasis (LNM) group and a nonlymph node metastasis (NLNM) group.

### Anthropometric measurements and history taking

All anthropometric measurements were performed on their first day of admission to our hospital. Each measurement was performed twice, and the mean value was then calculated. The history obtained from the patients included reproductive history, medical history, and family history of malignancy.

### Collection and storage of blood samples

On the morning of the operation, 5 mL of venous blood was drawn from each patient. The blood samples were delivered to the laboratory immediately for centrifugation. The serum samples were stored at −80°C for future analysis.

### Laboratory examinations

The serum samples were assayed at the clinical laboratory of Yantai Yuhuangding Hospital. The laboratory staff was blinded to the status of the patients. As the estrogen level of premenopausal women depends on the phase of the menstrual cycle, estrogen level was only determined in postmenopausal women. Serum insulin and estrogen concentrations were examined by radioimmunoassay (Siemens Medical Solutions Diagnostics, Los Angeles, USA) which demonstrated an interassay correlation variation [CV] of 6.0% and 5.6%, respectively. Serum C‐peptide level was measured using chemoluminescence (Siemens Healthcare Diagnostics Inc, Gwynedd, UK; interassay CV of 6.1%). Serum interleukin‐6 (IL‐6) level was measured by enzyme‐linked immunoassay (R&D Systems Europe, Lille, France; interassay CV of 9.2%). Serum tumor necrosis factor (TNF)‐*α* level was measured by a multiplex assay (Milliplex Human Adipokine Panel B, Millipore, Billerica, MA; interassay CV of 16.3%). Serum Ca‐125 level was measured with an electrochemiluminescence immunoassay with ADVIA CENTAUR XP (Siemens, Munich, Germany; interassay CV of 3.9%).

### Statistical analysis

SAS software package (Version 9, SAS Institute, Cary, NC) was used for data analysis. Serum estrogen, insulin, C‐peptide, CA125, TNF‐*α*, and IL‐6 levels were all natural logarithm‐transformed to normalize their distributions. Differences between groups were tested for continuous variables using the Student's *t*‐test and for categorical variables using the chi‐square test. Spearman correlation coefficients were calculated to evaluate the correlations between insulin and potential confounders. Univariate and multivariate logistic regression models were developed to estimate the associations between the variables and LNM risk. Serum insulin levels of pre‐ and postmenopausal patients were categorized into quartiles, with cut‐off points determined based on the distributions of the patients with NLNM. Likelihood ratio tests were performed to examine the linear trends in ORs with assigned quantitative scores, 1, 2, 3, and 4 for the categories. As obesity, particularly visceral obesity, is a well‐established risk factor for insulin resistance, BMI and WHR were adjusted for in our analysis. All *P* values were two‐sided among which lower than 0.05 is considered statistically significant.

## Results

### Basic characteristics of patients with lymph node metastases and nonlymph node metastases

A total of 668 patients were recruited into this study, whose baseline and pathological characteristics are shown in Table 1. Of these, 206 were premenopausal and 462 were postmenopausal. For the premenopausal cohort, 14 women showed evidence of LNM (7%), whereas the remaining 192 did not. Those with LNM had similar age (42.4, 95% confidence interval [CI]: 34.0–51.4 years vs. 41.8, 32.2–52.2 years; *P* > 0.05), a higher BMI (28.9, 22.7–34.7 kg/m^2^ vs. 24.4, 20.2–32.1 kg/m^2^; *P* < 0.001), and WHR (0.9, 0.8–1.0 vs. 0.8, 0.7–0.9; *P* < 0.001), but nevertheless similar proportions of high birthweight >4 kg (21% vs. 22%) and statistically indistinguishable age at menarche (14.0, 9.8–17.0 years vs. 13.6, 10.4–17.0 years) compared to those with NLNM. The incidences of diabetes (36% vs. 24%, *P* > 0.05) and hypertension (27% vs. 16%, *P* > 0.05) were not significantly different between both groups. However, the LNM group had a higher proportion of patients with lesion diameter >2 cm (57% vs. 19%; *P* < 0.01) and with myometrial invasion ≥50% (43% vs. 14%; *P* < 0.05). The LNM group also had a lower of proportion of patients with pathological grade 1 (29% vs. 81%; *P* < 0.001) and a higher proportion with pathological grade 2 (29% vs. 9%; *P* < 0.05) and 3 (43% vs. 10%; *P* < 0.01) lesions. Finally, patients with LNM had higher Ca‐125 (22.3, 9.4–322.8 U/mL vs. 14.3, 4.7–299.2 U/mL; *P* < 0.05) and insulin (11.0, 8.4–14.1 *μ*IU/mL vs. 7.9, 3.2–12.2 *μ*IU/mL; *P* < 0.01) levels but statistically indistinguishable CRP (1205, 1161–1488 ng/mL vs. 1291, 1029–1379 ng/mL; *P* > 0.05), TNF‐*α* (1.1, 0.9–1.7 pg/mL vs. 1.1, 0.7–1.8 pg/mL; *P* > 0.05), and IL‐6 (1.5, 1.1–1.7 pg/mL vs. 1.5, 1.2–1.7 pg/mL; *P* > 0.05) levels compared to those with NLNM.

**Table 1:** Clinical and pathological characteristics of LNM and NLNM endometrial cancer patients

	Premenopausal women			Postmenopausal women		
	LNM (*n* = 14)	NLNM (*n* = 192)	*P* value	LNM (*n* = 35)	NLNM (*n* = 427)	*P* value
Age	42.43 (34.01–51.39)	41.84 (32.17–52.18)	0.339	64.72 (49.51–76.02)	59.20 (50.05–74.67)	0.048
BMI (Kg/m^2^)	28.94 (22.73–34.69)	24.38 (20.19–32.09)	<0.001	28.15 (20.76–33.58)	25.01 (20.64–32.01)	<0.001
WHR	0.91 (0.80–0.99)	0.80 (0.69–0.93)	<0.001	0.88 (0.75–0.96)	0.81 (0.67–0.94)	<0.001
Birthweight >4 kg	3 (21.42)	42 (21.88)	0.969	9 (25.71)	104 (24.36)	0.083
Age at menarche	13.98 (9.81–17.01)	13.56 (10.44–17.03)	0.779	14.54 (10.00–15.09)	14.57 (10.52–16.06)	0.862
Age at menopause	–	–	–	51.73 (46.38–56.40)	50.50 (47.33–54.94)	0.997
Diabetes (%)	5 (35.71)	46 (23.96)	0.342	10 (28.57)	113 (26.46)	0.843
Hypertension (%)	4 (26.67)	30 (15.63)	0.278	13 (37.14)	201 (47.07)	0.293
Lesion diameter >2 cm (%)	8 (57.14)	36 (18.75)	0.003	17 (48.57)	66 (15.46)	<0.001
Myometrial invasion ≥50% (%)	6 (42.86)	27 (14.06)	0.013	16 (45.71)	39 (9.13)	0.002
Pathologic grade (%)						
1	4 (28.57)	155 (80.73)	<0.001	3 (8.57)	354 (82.90)	<0.001
2	4 (28.57)	18 (9.37)	0.048	9 (25.71)	55 (12.88)	0.043
3	6 (42.86)	19 (9.90)	0.003	23 (65.72)	18 (4.22)	<0.001
Ca‐125 (U/mL)	22.36 (9.42–322.84)	14.29 (4.73–299.17)	0.021	24.18 (7.46–376.33)	12.69 (3.34–215.64)	0.019
Estrogen (pg/mL)	–	–	–	23.97 (13.85–40.99)	21.01 (9.03–36.48)	0.061
Insulin (*μ*IU/mL)	11.01 (8.42–14.06)	7.91 (3.17–12.19)	0.008	10.59 (8.08–14.55)	7.13 (2.99–13.22)	0.011
CRP (ng/mL)	1205 (1161–1488)	1291 (1029–1379)	0.953	1176 (901–1354)	1125 (821–1466)	0.899
TNF‐*α* (pg/mL)	1.07 (0.88–1.69)	1.05 (0.67–1.78)	0.716	1.06 (0.79–1.69)	1.06 (0.55–1.57)	0.964
IL‐6 (pg/mL)	1.48 (1.11–1.69)	1.49 (1.22–1.70)	0.822	1.42 (1.09–1.77)	1.41 (1.01–2.13)	0.901

LNM, lymph node metastasis; NLNM, nonlymph node metastasis; BMI, body mass index; WHR, waist‐to‐hip ratio; CRP, C‐reactive protein.

Continuous variables are shown as means (95% CI), while categorical variables are shown as their positive percentages.

For the postmenopausal cohort, 35 women showed evidence of LNM (8%), whereas the remaining 427 did not. Those with LNM were older (64.7, 49.5–76.0 years vs. 59.2, 50.1–74.7 years; *P* < 0.05), had a higher BMI (28.2, 20.8–33.6 kg/m^2^ vs. 25.0, 20.6–32.0 kg/m^2^; *P* < 0.001) and WHR (0.9, 0.8–1.0 vs. 0.8, 0.7–0.9; *P* < 0.001) but nevertheless similar proportions of high birthweight >4 kg (25% vs. 24%; *P* > 0.05) and statistically indistinguishable age at menarche (14.5, 10.0–15.1 years vs. 14.6, 10.5–16.1 years; *P* > 0.05) and age at menopause (51.7, 46.4–56.4 years vs. 50.5, 47.3–54.9 years; *P* > 0.05) compared to those with NLNM. The incidences of diabetes (29% vs. 26%; *P* > 0.05) and hypertension (37% vs. 47%; *P* > 0.05) were not significantly different between both groups. However, the LNM group had a higher proportion of patients with lesion diameter >2 cm (49% vs. 15%; *P* < 0.001) and with myometrial invasion ≥50% (46% vs. 9%; *P* < 0.01). The LNM group also had a lower of proportion with pathological grade 1 (9% vs. 83%; *P* < 0.001) and a higher proportion with pathological grade 2 (26% vs. 13%; *P* < 0.05) and 3 (66% vs. 4%; *P* < 0.001) lesions. Finally, patients with LNM had higher Ca‐125 (24.2, 7.5–376.3 U/mL vs. 12.7, 3.3–215.6 U/mL; *P* < 0.05) and insulin (10.6, 8.1–14.6 *μ*IU/mL vs. 7.1, 3.0–13.2 *μ*IU/mL; *P* < 0.05) levels but statistically indistinguishable estrogen (24.0, 13.9–40.1 vs. 21.0, 9.0–36.5; *P* > 0.05), CRP (1176, 901–1354 ng/mL vs. 1125, 821–1466 ng/mL; *P* > 0.05), TNF‐*α* (1.1, 0.8–1.7 pg/mL vs. 1.1, 0.6–1.6 pg/mL; *P* > 0.05), and IL‐6 (1.4, 1.1–1.8 pg/mL vs. 1.4, 1.0–2.1 pg/mL; *P* > 0.05) levels compared to those with NLNM.

### Correlations between clinical or biochemical parameters and insulin levels in endometrial cancer patients

The correlations between clinical or biochemical parameters and insulin levels are shown in Table 2. In the LNM and NLNM groups of both premenopausal and postmenopausal women, insulin levels showed significant positive correlations with BMI and WHR but not with high birthweights, age at menarche, or age at menopause. Moreover, insulin levels also showed positive correlations with diabetes in premenopausal and postmenopausal LNM and NLNM groups, but the correlation coefficient for the postmenopausal LNM group did not reach statistical significance (*P* > 0.05). Insulin levels further showed positive correlations with hypertension in premenopausal and postmenopausal LNM groups, but not in NLNM groups. Insulin levels were significantly and positively correlated with greater disease severity of increased lesion diameter >2 cm, myometrial invasion ≥50%, and pathological grade. Finally, insulin levels positively correlated with estrogen levels, but not with Ca‐125, CRP, TNF‐*α*, or IL‐6 in any of the groups.

**Table 2:** Spearman correlation coefficients for associations between circulating insulin levels and the risk factors of lymph node metastasis

	Insulin (premenopausal women)		Insulin (postmenopausal women)	
	LNM (*n* = 14)	NLNM (*n* = 192)	LNM (*n* = 35)	NLNM (*n* = 427)
Age	0.05	0.09	0.19†	0.17
BMI	0.41‡	0.37‡	0.39‡	0.42‡
WHR	0.31‡	0.33‡	0.33‡	0.40‡
Birthweight >4 kg	0.11	0.15	0.16	0.14
Age at menarche	0.06	0.13	0.12	0.09
Age at menopause	–	–	0.14	0.12
Diabetes	0.18†	0.19†	0.17	0.19†
Hypertension	0.20†	0.16	0.22†	0.15
Lesion diameter >2 cm	0.24†	0.22†	0.27†	0.19†
Myometrial invasion ≥50%	0.29†	0.30†	0.31†	0.31†
Pathologic grade	0.33‡	0.32‡	0.30†	0.28†
Ca‐125	0.14	0.16	0.17	0.15
Estrogen	–	–	0.27†	0.25†
CRP	0.09	0.14	0.11	0.09
TNF‐*α*	0.11	0.12	0.13	0.10
IL‐6	0.15	0.14	0.14	0.13

LNM, lymph node metastasis; NLNM, nonlymph node metastasis; BMI, body mass index; WHR, waist‐to‐hip ratio; CRP, C‐reactive protein.

^†^ *P*<0.05; ^‡^ *P*<0.01 (two‐sided test).

### Predictors of lymph node metastases

Univariate logistic regression was used to examine the relationship between clinical or biochemical parameters and LNM risk. In premenopausal women (Table 3), BMI, WHR, lesion diameter >2 cm, myometrial invasion ≥50%, and pathological grade, but not age, high birthweight, age at menarche, diabetes, or hypertension, were associated with LNM risk. In terms of biochemical parameters, Ca‐125, and insulin, but not CRP, TNF‐*α* or IL‐6 predicted LNM risk. Stepwise multivariate logistic regression then demonstrated that only WHR, lesion diameter >2 cm, myometrial invasion ≥50%, pathological grade, and insulin levels remained significant predictors of LNM risk. The same parameters that showed significant predictive values for LNM risk in both univariate and multivariate analyses in premenopausal women were also predictive in postmenopausal women (Table 4).

**Table 3:** Odds ratio for lymph node metastasis risk among premenopausal endometrial cancer patients using univariate and multivariate logistic regressions

	Univariate logistic regression			Multivariate logistic regression		
	OR	95% CI	*P* value	OR	95% CI	*P* value
Age	0.88	0.52–1.28	0.195	0.75	0.44–1.20	0.223
BMI	2.28	1.79–2.80	0.031	1.51	0.93–1.90	0.059
WHR	4.94	2.52–11.56	0.003	2.59	1.36–3.81	0.035
Birthweight >4 kg	1.23	0.68–1.94	0.435	1.02	0.55–1.67	0.637
Age at menarche	1.51	0.58–1.98	0.657	1.07	0.47–1.64	0.258
Diabetes	1.88	0.72–2.86	0.297	1.16	0.42–1.82	0.452
Hypertension	1.54	0.65–2.30	0.095	1.22	0.64–1.71	0.106
Lesion diameter >2 cm	2.67	1.56–3.88	0.033	1.59	1.20–1.87	0.040
Myometrial invasion ≥50%	4.22	2.67–6.06	0.002	2.64	1.99–4.23	0.009
Pathological grade	4.30	2.77–6.84	0.004	2.43	1.95–3.16	0.008
Ca‐125	2.17	1.20–2.89	0.038	1.84	0.97–3.10	0.057
Insulin	3.32	1.88–4.69	0.007	2.11	1.48–2.85	0.023
CRP	1.88	0.94–2.79	0.089	1.09	0.74–1.51	0.196
TNF‐*α*	1.96	0.90–3.21	0.079	1.28	0.75–1.92	0.109
IL‐6	1.53	0.76–2.46	0.081	1.06	0.46–1.72	0.202

**Table 4:** Odds ratio for lymph node metastasis risk among postmenopausal endometrial cancer patients using univariate and multivariate logistic regressions

	Univariate logistic regression			Multivariate logistic regression		
	OR	95% CI	*P* value	OR	95% CI	*P* value
Age	0.97	0.49–1.58	0.099	0.86	0.56–1.29	0.218
BMI	2.72	1.29–5.01	0.039	1.62	0.93–2.46	0.054
WHR	5.07	2.33–7.63	0.002	2.21	1.29–3.64	0.022
Birthweight >4 kg	1.43	0.79–1.64	0.204	1.11	0.64–1.76	0.532
Age at menarche	1.21	0.39–2.06	0.752	0.94	0.30–1.61	0.737
Age at menopause	1.34	0.46–2.39	0.684	0.85	0.24–1.55	0.691
Diabetes	1.90	0.81–3.21	0.167	1.34	0.64–2.20	0.533
Hypertension	1.57	0.71–2.24	0.678	1.11	0.55–1.83	0.672
Lesion diameter >2 cm	2.44	1.51–3.56	0.019	1.68	1.37–3.09	0.038
Myometrial invasion ≥50%	4.98	2.97–6.84	0.011	2.41	1.39–3.62	0.027
Pathologic grade	5.30	2.47–8.20	<0.001	2.52	1.77–3.23	0.012
Ca‐125	2.04	1.31–3.33	0.031	1.71	0.98–2.85	0.052
Estrogen	1.85	0.97–2.94	0.054	1.29	0.78–1.65	0.072
Insulin	3.91	2.06–5.34	<0.001	1.99	1.30–2.89	0.029
CRP	1.34	0.64–1.96	0.093	1.01	0.52–1.68	0.124
TNF‐*α*	1.53	0.76–2.22	0.097	1.08	0.67–1.55	0.089
IL‐6	1.34	0.71–2.03	0.073	0.97	0.51–1.45	0.103

Subsequent analyses involved the categorization of insulin levels into quartiles. In premenopausal women (Table 5), compared to the first quartile, there was a dose‐dependent relationship between insulin levels and LNM risk with odds ratios of 1.51 (95% CI: 0.90–2.49; *P* > 0.05), 2.23 (1.34–3.39; *P* < 0.05), and 4.88 (2.26–7.05; *P* < 0.05) for the second, third, and fourth quartiles, respectively. However, only quartile four (cut‐off: 10.48 *μ*IU/mL) remained predictive of LNM risk when adjusted for BMI (OR: 3.51, 1.42–5.98; *P* < 0.05) or for WHR (OR: 1.87, 1.08–2.66; *P* < 0.05). In postmenopausal women (Table 6), compared to the first quartile, there was a dose‐dependent relationship between insulin levels and LNM risk with odds ratios of 1.49 (95% CI: 0.80–2.38; *P* > 0.05), 2.19 (1.26–3.44; *P* < 0.05), and 4.18 (2.37–6.66; *P* < 0.05) for the second, third, and fourth quartiles, respectively. However, only quartiles three and four (cut‐off: 7.40 and 10.15 *μ*IU/mL, respectively) remained predictive of LNM risk when adjusted for BMI (OR: 1.99, 1.01–3.12, *P* < 0.05 and 3.07, 1.26–5.40; *P* < 0.05, respectively), and only quartile four was predictive of LNM risk when adjusted for WHR (OR: 1.61, 1.04–2.35; *P* < 0.05).

**Table 5:** Risk (odds ratio, OR (95% confidence interval, CI)) of lymph node metastasis risk among premenopausal endometrial cancer patients by categories of insulin

	Categories				*P*_trend_
	1	2	3	4	
Quartile cut‐offs (*μ*IU/mL)	<5.56	5.56–7.64	7.65–10.48	>10.48	
Crude OR	1	1.51 (0.90–2.49)	2.23 (1.34–3.39)	4.88 (2.26–7.05)	0.034
Adjusted for BMI	1	1.34 (0.66–2.19)	2.04 (0.95–3.26)	3.51 (1.42–5.98)	0.042
Adjusted for WHR	1	1.02 (0.52–1.65)	1.89 (0.84–2.86)	1.87 (1.08–2.66)	0.048

*P* value for trend with assigned quantitative scores 1, 2, 3, and 4 for the categories.

Cut‐off points were based on the distribution of the nonlymph node metastasis premenopausal patients.

**Table 6:** Risk (odds ratio, OR (95% confidence interval, CI)) of lymph node metastasis risk among postmenopausal endometrial cancer patients by categories of insulin

	Categories				*P*_trend_
	1	2	3	4	
Quartile cut‐offs (*μ*IU/mL)	<5.14	5.14–7.39	7.40–10.15	>10.15	
Crude OR	1	1.49 (0.80–2.38)	2.19 (1.26–3.44)	4.18 (2.37–6.66)	0.037
Adjusted for BMI	1	1.23 (0.49–2.02)	1.99 (1.01–3.12)	3.07 (1.26–5.40)	0.044
Adjusted for WHR	1	0.99 (0.43–1.61)	1.47 (0.64–2.62)	1.61 (1.04–2.35)	0.047

*P* value for trend with assigned quantitative scores 1, 2, 3, and 4 for the categories.

Cut‐off points were based on the distribution of the nonlymph node metastasis postmenopausal patients.

## Discussion

The main findings of this study are that (1) WHR, lesion diameter >2 cm, myometrial invasion ≥50%, pathological grade, and insulin levels were significant predictors of LNM risk in both premenopausal and postmenopausal women, (2) insulin level with a cut‐off of 10.48 *μ*IU/mL was predictive of LNM risk when adjusted for BMI (OR: 3.51, 1.42–5.98; *P* < 0.05) or for WHR (OR: 1.87, 1.08–2.66; *P* < 0.05) in premenopausal women, and (3) insulin with a similar cut‐off of 10.15 *μ*IU/mL was predictive of LNM risk when adjusted for BMI (3.07, 1.26–5.40; *P* < 0.05, respectively) or for WHR (OR: 1.61, 1.04–2.35; *P* < 0.05).

In our previous case–control study conducted in China 21, we identified insulin as an independent predictor of endometrial cancer in premenopausal women. Similarly, a case–control study involving a Caucasian population in Alberta, Canada, also found that the highest quartile of insulin, in comparison with the lowest quartile, was associated with an increased risk of endometrial cancer 22. In another prospective cohort study, insulin levels, comparing the highest with the lowest quartile, were associated with a twofold increase in the risk of endometrial cancer in 93,676 postmenopausal women 23. We extend these findings by demonstrating that insulin was an independent predictor of LNM risk in endometrial cancer for both premenopausal and postmenopausal Chinese women.

Epidemiological studies have shed insights into a possible mechanistic link between insulin and the risk of endometrial cancer 24, which includes sex steroid hormones, adipokines, and low‐grade inflammation. Firstly, endometrial cancer is an estrogen‐dependent cancer 25 and as aforementioned, insulin can inhibit the synthesis of sex hormone‐binding globulin (SHBG) that usually binds to steroid hormones 26, leading to increased free estrogen levels in the blood. Estrogen exerts its effects by stimulating the ER*α*, ER*β*, or GPER (G protein‐coupled estrogen receptor 1) receptors, leading to a proliferative response in the endometrium 27. Secondly, as also stated previously, insulin may act independently via its downstream PI3K/AKT and Ras/MAPK signaling pathways 28 to induce cell proliferation and inhibit apoptosis, thereby promoting endometrial cancer development. Thirdly, insulin can increase the circulating levels of insulin growth factor‐1 29, which has been linked to an increased proliferation of the endometrium. Fourthly, increased insulin levels are associated with obesity, and several adipokines have in turn been implicated in the disease pathogenesis of endometrial cancer. Thus, in a case–control study within the Prostate, Lung, Colorectal, and Ovarian Cancer (PLCO) Screening Trial, the relationship of prediagnostic serum levels of adiponectin, leptin, and visfatin with postmenopausal endometrial cancer risk was prospectively evaluated 30. This demonstrated that higher leptin levels were associated with higher cancer risk, whereas higher adiponectin levels and adiponectin‐to‐leptin ratio were associated with a lower risk. Finally, there is a relationship between insulin levels, insulin resistance, chronic inflammation, and endometrial cancer risk 31, 32. Low‐grade inflammation can promote the neoplastic transformation of endometrial tissue via several mechanisms, namely by inducing the generation of reactive oxygen species, which causes DNA mutations and increased cell proliferation 33, 34, as well as by mediating the dysregulation of the NF‐*κ*B pathway, leading to the inhibition apoptosis 35.

## Conclusion

In conclusion, this epidemiological study demonstrated insulin to be an independent predictor of LNM. Further prospective studies are needed to confirm this association and its role as a biomarker for determining clinical outcomes and prognosis. Finally, basic science studies are needed to elucidate the mechanistic pathways that potentially explain a causal relationship between insulin levels and cancer metastasis in endometrial cancer.

## Conflict of Interest

None declared.
